# Current situation and needs analysis of medical staff first aid ability in China: a cross-sectional study

**DOI:** 10.1186/s12873-023-00891-x

**Published:** 2023-11-03

**Authors:** Juntao Wang, Chuanzhu Lv, Xingyue Song, Yanlan Hu, Wenjie Hao, Lanfen He, Yu Chen, Yong Gan, Xiaotong Han, Shijiao Yan

**Affiliations:** 1 International School of Public Health and One Health, Hainan Medical University, Haikou, Hainan China; 2 Emergency Medicine Center, Sichuan Provincial People’s Hospital, University of Electronic Science and Technology of China, Chengdu, Sichuan China; 3 Research Unit of Island Emergency Medicine, Chinese Academy of Medical Sciences (No. 2019RU013), Hainan Medical University, Haikou, Hainan China; 4 Key Laboratory of Emergency and Trauma of Ministry of Education, Hainan Medical University, Haikou, Hainan China; 5 Department of Emergency, Hainan Clinical Research Center for Acute and Critical Diseases, The Second Affiliated Hospital of Hainan Medical University, Haikou, Hainan China; 6 Department of Social Medicine and Health Management, School of Public Health, Tongji Medical College, Huazhong University of Science and Technology, Wuhan, Hubei China; 7 Department of Emergency Medicine, Hunan Provincial Key Laboratory of Emergency and Critical Care Metabolomics, Hunan Provincial Institute of Emergency Medicine, Hunan Provincial People’s Hospital/The First Affiliated Hospital, Hunan Normal University, Changsha, Hunan China

**Keywords:** Cross-sectional study, Hospital level, Medical staff, First aid training, China

## Abstract

**Objectives:**

We aim to understand the current situation of the first aid ability and training needs of Chinese medical personnel to provide a scientific basis for formulating the contents and methods of emergency medical rescue training and thereby improve the first aid level of Chinese medical personnel.

**Methods:**

A cross-sectional survey was conducted between June 2022 and February 2023 using a two-stage cluster sampling method with a structured questionnaire sent to medical workers in 12 provinces in China. 14,527 questionnaires were included in this study. Data were collected on demographic characteristics, first aid knowledge and skills, and training needs. Variance analysis was used to compare the difference between the first aid ability and training needs of medical staff in different hospitals, and multiple linear regression analysis was carried out to evaluate first aid ability and training needs.

**Result:**

The study included 6041 patients (41.6%) in tertiary hospitals, 5838 patients (40.2%) in secondary hospitals, and 2648 patients (18.2%) in primary hospitals. There were significant differences in the first aid ability and training needs of medical staff in hospitals of different levels (*p* < 0.001). The score of first aid knowledge and skills in tertiary hospitals was the highest (209.7 ± 45.0), and the score of training needs in primary hospitals was the highest (240.6 ± 44.0). There was a significant correlation between first aid ability and training needs score (*p* < 0.001). Multiple linear regression analysis shows that geographic region, age, work tenure, gender, job title, department, professional title, monthly income, and hospital level are the influencing factors of training demand.

**Conclusion:**

Medical staff in primary hospitals generally have low first aid knowledge and skills and a strong willingness to train. Therefore, it is imperative to strengthen the training of first aid ability and research training strategies. The level of the hospital is closely related to the level of first aid, so it is necessary to recognize the commonalities and differences in medical staff’s demand for first aid knowledge and skills and carry out targeted education and training.

**Supplementary Information:**

The online version contains supplementary material available at 10.1186/s12873-023-00891-x.

## Introduction

The emergency medical service system is a critical component of the public health emergency response system [1]. As the first line of health defence in response to public health emergencies, medical personnel’s first aid ability is very important to improve the treatment quality and success rate for all kinds of urgent and critically ill patients. Scholars from the United States, the United Kingdom, Switzerland, and other countries have found that the improvement in the emergency treatment ability of hospital medical staff can significantly reduce emergency room mortality [2, 3].

In the 1950s, with the help of the World Health Organization (WHO), many countries explored and practiced the division of medical resources to varying degrees [4, 5]. According to the function of medical institutions and the scope of diagnosis and treatment, China divides the public medical institutions into three levels [6]. The primary hospitals are mainly township hospitals and community health service centers, which mainly provide primary medical services. The secondary hospital is a multi-community medical service institution that deal with common diseases that are difficult for primary care institutions to solve and provide referral services. A tertiary hospital is the highest grade and is responsible for providing a high level of acute and critical care. Due to the differences in internal management, functional division of labor, talent reserve and other aspects of hospitals at different levels, there are also differences in first-aid ability of medical staff in hospital [7, 8]. However, with rapid socioeconomic growth and the significant increase in the demand for quality medical resources, and the diagnosis and treatment ability of primary medical staff is often difficult to meet the needs of the public and society [9]. The contradiction between people’s growing demand for first aid and unreasonable allocation of first aid resources is gradually deepening. Therefore, it is critical to train medical personnel in China who are suitable for the current situation of China’s medical service system and have solid knowledge of emergency medicine and basic skills of first aid.

Research has shown that first aid training is a highly cost-effective way to reduce mortality from acute and critical illnesses and improve global health [9]. Teaching medical staff knowledge and techniques of relevant first aid and equipping them to use them is an important factor in saving the lives of patients. Medical staff from different disciplinary backgrounds and different levels of hospitals show different degrees of understanding in first aid training [10]. The content and methods of education and training need to be designed in a targeted manner according to the characteristics and needs of the medical staff at all levels of medical institutions. However, the current research on the status of medical workers’ first aid ability and training needs is limited [11–13]. Therefore, we conducted a large-scale cross-sectional study in 12 Chinese provinces to understand the current situation and training needs of medical staff in China and provide a theoretical basis for designing the content and methods of first aid education and training to improve the level of first aid ability of medical staff, minimize patient suffering and save patient lives.

## Materials and methods

### Study design and participants

**Fig. 1:**
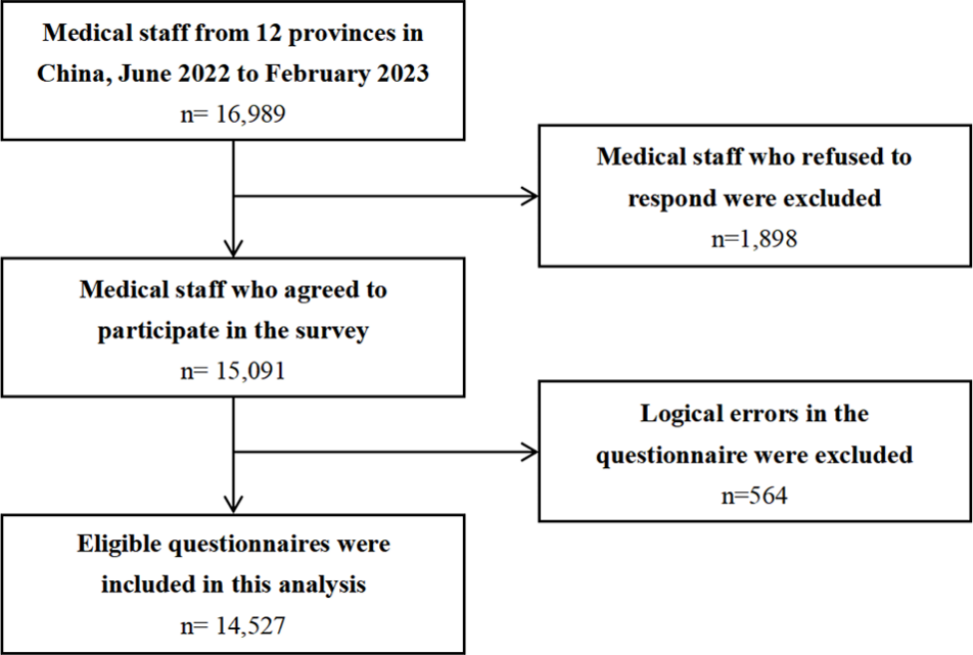
The flowchart of study participants

A cross-sectional study was conducted in China from June 2022 to February 2023. The study used a two-stage cluster sampling strategy to obtain the study sample. First, 12 provinces/municipalities/autonomous regions were selected: Hubei, Hunan, Guangdong, Guangxi, Hainan, Zhejiang, Fujian, Jiangxi, Sichuan, Guizhou, Yunnan, and Chongqing. The 12 provinces were divided into eastern, central, and western China according to their economic levels. The convenience sampling method was then used to select 50 to 80 health institutions in each province, and all medical personnel in each hospital were selected as a group. The questionnaire was designed and administered using the Chinese online survey tool Questionnaire Star (https://www.wjx.cn/), and a structured online questionnaire was distributed via WeChat to collect data. Questionnaires were distributed by hospital managers to medical staff in the hospital, limiting each device to only one questionnaire to ensure the accuracy and validity of the data. A total of 15,091 medical workers agreed to participate in the survey, and 564 questionnaires were excluded due to logical errors. The final 14,527 (96.3%) questionnaires were included in the study (Fig. 1).

### Ethics statement

The study protocol was approved by the Medical Ethics Committee of Hainan Medical College (HYLL-2022-226). Participants’ information is completely confidential, questionnaires are recorded anonymously, and survey contents are used only for scientific research.

### Questionnaire design and definition

The questionnaire was designed using a literature review, group discussion, and expert panel discussion. The questionnaire is divided into three sections: (1) Participants’ demographic data, such as gender, age, and educational level; (2) Participants’ basic first aid knowledge. A total of 37 conventional first aid theoretical knowledge questions and 19 single first aid operation skill items were constructed [14]. (3) Participants’ demand for first aid training. The questionnaire findings were scored on a 5-point Likert-type scale ranging from 1 (very poor) to 5 (very good), with higher scores indicating stronger ability or willingness. The Cronbach’s α value of the questionnaire scale was 0.983, and the KMO measure of sampling adequacy was 0.991. The result of Bartlett’s test of sphericity was significant (*p* < 0.05), indicating that the questionnaire had good reliability and validity (Supplementary File).

### Statistical analysis

Microsoft Excel 2016 was used to summarize the questionnaires that were collected. SPSS 22.0 was used to analyse the data. The descriptive statistics of categorical variables were summarized as percentages and frequencies, and the chi-square (χ^2^) test was used to calculate associations between categorical variables. A descriptive analysis of continuous variables was conducted using the mean ± standard deviation. When the homogeneity test of variance was significant, the Welch test was used to compare the differences in first-aid ability and training needs of medical staff at different levels of hospitals, and the Games-Howell test was used for pairwise comparison between groups. Spearman’s correlation and multiple linear regression analyses were performed on the first aid competency and training need scores. Bilateral tests were used for all significance level tests, and *p* < 0.05 was considered statistically significant.

## Results

### Participant characteristics

A total of 14,527 subjects were included in our study. The majority of subjects were in tertiary hospitals (41.6%), in central China (38.2%), aged between 31 and 40 years old (37.7%), with work tenure ≤ 10 years (56.8%), female (57.4%), doctors (53.0%), with a bachelor’s degree (62.3%), in the emergency department (44.2%), with a professional title at the primary level or below (54.8%), and with monthly income < 8000 (80.1%). The results of the χ^2^ test showed that there were significant differences in the composition of geographical region, age, working years, gender, occupation type, education level, department, professional title, and monthly income of medical staff at different levels of hospitals (*p* < 0.001). The characteristics of the participants are shown in Table 1.

**Table 1:** Descriptive statistics on general information of medical staff in different levels of hospitals

Variables	Total n (%)	Hospital level			*x* ^2^	*P* value
		Tertiary	Secondary	Primary		
**Total n (%)**	14,527(100)	6041(41.6)	5838(40.2)	2648(18.2)		
**Geographic region**						
Eastern China	4170(28.7)	1972(31.9)	1623(27.8)	620(23.4)	880.52	< 0.001
Central China	5549(38.2)	1492(24.7)	2769(47.4)	1288(48.6)		
Western China	4808(33.1)	2622(43.4)	1446(24.8)	740(27.9)		
**Age**						
≤ 30	5384(37.1)	2154(35.7)	2289(39.2)	941(35.5)	214.94	< 0.001
31–40	5482(37.7)	2574(42.6)	2113(36.2)	795(30.0)		
≥ 41	3661(25.2)	1313(21.7)	1436(24.6)	912(34.4)		
**Work tenure**						
≤ 10	8250(56.8)	3553(58.8)	3346(57.3)	1351(51.0)	46.68	< 0.001
> 10	6277(43.2)	2488(41.2)	2492(42.7)	1297(49.0)		
**Gender**						
Male	6183(42.6)	2910(48.2)	2349(40.2)	924(34.9)	154.34	< 0.001
Female	8344(57.4)	3131(51.8)	3489(59.8)	1724(65.1)		
**Job title**						
Doctors	7698(53.0)	3314(54.9)	3014(51.6)	1370(51.7)	614.68	< 0.001
Nures	6051(41.7)	2637(43.7)	2514(43.1)	900(34.0)		
Others	778(5.4)	90(1.5)	310(5.3)	378(14.3)		
**Education level**						
Associate’s degree or vocational diploma	4453(30.7)	1151(19.1)	1738(29.8)	1564(59.1)	1856.30	< 0.001
Bachelor degree	9051(62.3)	4072(67.4)	3925(67.2)	1054(39.8)		
Master degree or higher	1023(7.0)	818(13.5)	175(3.0)	30(1.1)		
**Department**						
Emergency	6418(44.2)	4412(73.0)	1857(31.8)	149(5.6)	5588.69	< 0.001
general surgery	1237(8.5)	399(6.6)	674(11.5)	164(6.2)		
General medicine	2012(13.9)	286(4.7)	544(9.3)	1182(44.6)		
Others	4860(33.5)	944(15.6)	2763(47.3)	1153(43.5)		
**Professional title**						
Elementary or below	7960(54.8)	3050(50.5)	2989(51.2)	1921(72.5)	434.64	< 0.001
Intermediate	4444(30.6)	1950(32.3)	1945(33.3)	549(20.7)		
Senior	2123(14.6)	1041(17.2)	904(15.5)	178(6.7)		
**Monthly income (¥)**						
< 3000	1795(12.4)	367(6.1)	622(10.7)	806(30.4)	2730.71	< 0.001
3000–5000	5314(36.6)	1486(24.6)	2439(41.8)	1389(52.5)		
5001–8000	4522(31.1)	2165(35.8)	1987(34.0)	370(14.0)		
≥ 8001	2896(19.9)	2023(33.5)	790(13.5)	83(3.1)		

### Analysis of participants’ first aid ability

The evaluation of first aid ability included 2 scales of first aid knowledge and single first aid skill mastery. First aid knowledge includes 37 questions with a total score of 185, mainly divided into 10 aspects: first aid overview, inspection classification, trauma emergency, environmental emergency, circulatory emergency, respiratory emergency, gastrointestinal emergency, endocrine emergency, neurological emergency, and other emergencies. The individual skills included 19 topics with a total score of 95, mainly divided into 14 aspects of cardiopulmonary resuscitation (CPR), airway obstruction removal, adult tracheal intubation, cricothyroid puncture, electrical defibrillation, electrical resuscitation, deep venipuncture, four major punctures, closed thoracic drainage, pericardiocentesis, war wound technique, dressing change, clear planning and suture, arterial puncture, and electrocardiographic (ECG) monitoring and recognition (Table 2). The results showed that the medical personnel had mastered the theoretical knowledge of first aid, with higher emergency treatment scores for hypoglycaemia (3.90 ± 1.09), allergic reaction (3.85 ± 1.20), shock (3.81 ± 1.08), transfusion reaction (3.73 ± 1.13), and hypertensive crisis (3.71 ± 1.10). The top 5 single emergency skills scores were adult CPR (4.01 ± 1.17), adult airway foreign body obstruction removal (3.59 ± 1.21), dressing change, clear planning and suture (3.49 ± 1.32), war wound technique (3.47 ± 1.30), and infant CPR (3.46 ± 1.17).

**Table 2:** Score of first aid knowledge and single first aid skill scale of medical staff

Scale	Variables(N = 56)	Score	Total (n = 14,527)	Hospital classification			Statistical values (*F*)	*P* value
				Tertiary	Secondary	Primary		
**First aid knowledge mastery scales**	37	185	133.14 ± 35.07	144.40 ± 31.47	131.53 ± 33.92	111.98 ± 34.12	947.77	< 0.001
First aid overview	1	5	3.60 ± 1.10	3.91 ± 1.03	3.54 ± 1.08	3.04 ± 1.05	652.57	< 0.001
Inspection classification	1	5	3.47 ± 1.14	3.84 ± 1.04	3.38 ± 1.12	2.86 ± 1.09	810.91	< 0.001
Emergency trauma	5	25	17.61 ± 5.19	19.20 ± 4.77	17.20 ± 5.10	14.90 ± 5.00	741.17	< 0.001
Environmental emergency	3	15	10.52 ± 2.91	11.24 ± 2.71	10.35 ± 2.90	9.23 ± 2.89	487.63	< 0.001
Circulating emergency	5	25	18.53 ± 5.10	19.98 ± 4.61	18.38 ± 5.00	15.57 ± 5.05	751.20	< 0.001
Respiratory emergency	4	20	14.29 ± 4.15	15.59 ± 3.74	14.12 ± 4.03	11.67 ± 3.99	938.29	< 0.001
Gastrointestinal emergency	4	20	14.47 ± 4.17	15.76 ± 3.37	14.27 ± 4.08	11.94 ± 4.08	870.13	< 0.001
Endocrine emergency	4	20	14.47 ± 4.05	15.68 ± 3.63	14.36 ± 3.67	11.94 ± 3.92	889.52	< 0.001
Nervous system emergency	5	25	18.10 ± 5.29	19.75 ± 4.74	17.91 ± 5.17	14.76 ± 5.10	935.68	< 0.001
Other emergency	5	25	18.08 ± 4.97	19.46 ± 4.49	18.02 ± 4.84	15.06 ± 4.96	770.14	< 0.001
**Single skill mastery scales**	19	95	59.17 ± 19.45	65.33 ± 18.47	58.43 ± 18.64	46.73 ± 16.99	1046.52	< 0.001
CPR	2	10	7.47 ± 2.15	7.91 ± 2.03	7.47 ± 2.10	6.48 ± 2.21	429.55	< 0.001
Airway obstruction removal	2	10	6.97 ± 2.31	7.57 ± 2.15	6.93 ± 2.24	5.72 ± 2.28	645.06	< 0.001
Adult tracheal intubation	1	5	3.09 ± 1.41	3.57 ± 1.35	2.99 ± 1.38	2.24 ± 1.15	1107.57	< 0.001
Cricothyroid puncture	1	5	2.68 ± 1.31	3.02 ± 1.32	2.62 ± 1.29	2.04 ± 1.07	675.91	< 0.001
Electrical defibrillation	1	5	3.43 ± 1.35	3.93 ± 1.19	3.39 ± 1.31	2.40 ± 1.18	1524.47	< 0.001
Electrical resuscitation	1	5	3.19 ± 1.35	3.69 ± 1.23	3.11 ± 1.31	2.22 ± 1.12	1497.18	< 0.001
Deep veinpuncture	1	5	2.72 ± 1.39	3.06 ± 1.42	2.67 ± 1.35	2.07 ± 1.12	600.35	< 0.001
Four major puncture	4	20	11.12 ± 5.38	12.33 ± 5.51	11.05 ± 5.25	8.54 ± 4.35	595.02	< 0.001
Closed thoracic drainage	1	5	2.76 ± 1.41	3.07 ± 1.44	2.73 ± 1.38	2.09 ± 1.15	577.25	< 0.001
pericardiocentesis	1	5	2.34 ± 1.28	2.59 ± 1.33	2.31 ± 1.26	1.84 ± 1.02	410.12	< 0.001
war wound technique	1	5	3.47 ± 1.30	3.77 ± 1.22	3.40 ± 1.29	2.94 ± 1.30	404.87	< 0.001
Dressing change, clear planing and suture	1	5	3.49 ± 1.32	3.66 ± 1.30	3.46 ± 1.31	3.19 ± 1.35	118.88	< 0.001
Arterial puncture	1	5	2.98 ± 1.39	3.41 ± 1.34	2.89 ± 1.37	2.20 ± 1.19	881.68	< 0.001
ECG monitoring and recognition	1	5	3.44 ± 1.26	3.77 ± 1.14	3.42 ± 1.25	2.76 ± 1.24	644.00	< 0.001

Kendall’s coefficient of rank correlation tau-sub-b was used to examine the association between subjects’ general information (ordinal variable) and first aid knowledge and skill scores (continuous variable). The results showed that subjects’ general information was correlated with both first aid knowledge and skill scores (*p* < 0.001). Geographic location, age, years of experience, gender, type of occupation, education, department, title, monthly income, and hospital grade were statistically significantly (*p* < 0.05) related to first aid knowledge and skill scores (Supplementary Table 2).

Levene’s test showed a lack of homogeneity of variance, so a corrected univariate analysis of variance must be used. Welch analysis of variance was used in our study. The results showed that the first-aid ability scores of medical staff in different hospitals had statistical significance (Welch F = 1170.31, *p* < 0.001). The Games-Howell test was used for pairwise comparison between groups. The results showed that the mean score of medical staff emergency competency was 32.24 points lower in primary hospitals than in secondary hospitals (95% CI: -34.85 to -29.63, *p* < 0.001). The mean score of secondary hospitals was 19.78 points lower than that of tertiary hospitals (95% CI: -21.79 to -29.63, *p* < 0.001). All differences were statistically significant (Table 3).

**Table 3:** Multiple comparison of first-aid ability and training needs of medical staff in different levels of hospitals

	Group(*I*)	Group(*J*)	Mean Difference(*I-J*)	S.E	*P* value	95% CI	
						Low	Up
First Aid Ability	Tertiary	Primary	52.02	1.089	< 0.001	49.88	54.16
	Secondary	Tertiary	-19.78	0.857	< 0.001	-21.79	-17.77
	Primary	Secondary	-32.24	1.113	< 0.001	-34.85	-29.63
Training Needs	Tertiary	Primary	-8.95	1.062	< 0.001	-11.44	-6.46
	Secondary	Tertiary	3.11	0.863	0.001	1.09	5.13
	Primary	Secondary	5.84	1.039	< 0.001	3.40	8.28

### Analysis of participants’ first aid training needs

The results showed that the top 5 theoretical pieces of training considered “very necessary” by medical personnel were emergency management of allergic reactions (42.58%), hypoglycaemia (42.19%), shock (42.19%), water-electrolyte acid-base imbalance (42.1%), and myocardial infarction (42.03%). The single operation skills were mainly CPR (46.06%), airway obstruction removal (45.78%), and ECG monitoring and recognition (44.95%) (Supplementary Table 2).

The first aid training needs scale scores are shown in Table 4. The willingness to learn theoretical knowledge of first aid (157.7 ± 41.1) and individual first aid operation skills (81.4 ± 16.8) was higher among medical personnel in primary hospitals than in secondary and tertiary hospitals. The demand for theoretical knowledge of emergency management of circulatory emergencies, endocrine emergencies, allergic reactions, and water-electrolyte acid-base imbalance was higher in primary hospitals. For single emergency skills, the demand for CPR, airway obstruction removal, and trauma management was higher (Supplementary Table 3).

**Table 4:** Scores of first-aid knowledge and skills and first-aid training requirements in hospitals of different levels

Scale	Scores (*Mean ± SD*)			Statistical values (*F*)	*P* value
	Tertiary	Secondary	Primary		
**First Aid Capability**					
Total	209.7 ± 45.0	190.0 ± 48.3	157.7 ± 41.1	1170.31	< 0.001
first aid knowledge	144.4 ± 31.5	131.5 ± 33.9	111.0 ± 34.1	947.77	< 0.001
Single first aid skills	65.3 ± 18.5	58.4 ± 18.6	46.7 ± 17.0	1046.52	< 0.001
**Training Needs**					
Total	231.7 ± 48.9	234.8 ± 45.1	240.6 ± 44.0	35.52	< 0.001
first aid knowledge	153.1 ± 34.2	155.1 ± 31.3	159.2 ± 30.3	35.49	< 0.001
Single first aid skills	78.6 ± 17.6	79.7 ± 16.3	81.4 ± 16.8	26.36	< 0.001

### Analysis of factors influencing first aid competency and training needs

Spearman’s correlation coefficient showed that there was a weak positive correlation (Spearman’s rho = 0.177) and a significant correlation (*p* < 0.001) between the first aid ability score and training needs score. As the first aid competency score increased, the willingness to train also increased (Table 5).

**Table 5:** Correlation between first aid competence and willingness to train among Chinese medical personnel (n = 14,527). Test applied: Spearman’s correlation test

First Aid Capability - Training Needs	Total		first aid knowledge		Single first aid skills	
	Spearman’s Coefficient Value (rho)	*P* value	Spearman’s Coefficient Value (rho)	*P* value	Spearman’s Coefficient Value (rho)	*P* value
Total	0.177	<0.001	0.240	<0.001	0.043	<0.001
Primary	0.027	0.165	0.703	< 0.001	-0.042	0.029
Secondary	0.221	< 0.001	0.276	<0.001	0.087	<0.001
Tertiary	0.285	< 0.001	0.362	<0.001	0.103	<0.001

The results of multiple linear regression analysis of first aid ability and training demand predictors are shown in Table 6. Multiple regression analysis was conducted with first aid knowledge and skill score and training demand score as dependent variables and general characteristics as independent variables. The regression tolerance was greater than 0.1, and the models had statistical significance (F_1_ = 632.793, R_1_^2^ = 30.4%, *p*_*1*_ < 0.001; F_2_ = 64.175, R_2_^2^ = 4.2%, *p*_*2*_ < 0.001). Scores of first aid knowledge and skill were correlated with geographic region (β = 0.047, *p* < 0.001), age (β = -0.074, *p* < 0.001), work tenure (β = 0.081, *p* < 0.001), gender (β = -0.043, *p* < 0.001), job title (β = -0.210, *p* < 0.001), department (β = -0.247, *p* < 0.001), professional title (β = 0.080, *p* < 0.001), monthly income (β = 0.104, *p* < 0.001) and hospital level (β = -0.167, *p* < 0.001). Among the 10 independent variables included in the model, all of them except the department (β = -0.104, *p* = 0.753) had statistical significance on the training demand score.

**Table 6:** Multiple linear regression Predicting first aid capability and willingness to train

Variables	First aid knowledge and skills score				Training Needs Scoring			
	*B*	*S.E*	*Beta*	*P* value	*B*	*S.E*	*Beta*	*P* value
(Constant)	235.915	3.000		< 0.001	205.933	3.26		< 0.001
Geographic region	3.009	0.450	0.047	< 0.001	9.312	0.489	0.157	< 0.001
Age	-4.767	1.075	-0.074	< 0.001	-1.657	0.782	-0.028	0.034
Work tenure	8.177	1.075	0.081	< 0.001	8.023	1.169	0.085	< 0.001
Gender	-4.347	0.838	-0.043	< 0.001	7.620	0.911	0.081	< 0.001
Job title	-17.693	0.714	-0.210	< 0.001	-6.462	0.776	-0.083	< 0.001
Education level	0.171	0.720	0.002	0.812	-2.892	0.783	-0.035	< 0.001
Department	-9.292	0.304	-0.247	< 0.001	-0.104	0.330	-0.003	0.753
Professional title	5.533	0.677	0.080	< 0.001	-2.986	0.736	-0.047	< 0.001
Monthly income	5.526	0.486	0.104	< 0.001	0.872	0.528	0.018	0.099
Hospital level	-11.410	0.585	-0.167	< 0.001	3.927	0.636	0.062	< 0.001

## Discussion

Our research mainly describes the differences in the first aid capabilities and training needs of medical staff among different levels of medical institutions in China. The correlation and influencing factors of first aid ability and training demand were analyzed. The research results provide theoretical basis for China to formulate the content of emergency medical rescue training scientifically.

In this large population cross-section study of 14,527 medical staff, we found that there are more medical staff with a master’s degree or above (79.9%), senior professional title (49.0%), and monthly income > 8000 (69.9%) in tertiary hospitals than in secondary and primary hospitals. The overall medical staff of secondary hospitals was younger (39.2% age ≤ 30). The ageing of the medical staff in primary hospitals is serious, with 44.9% aged > 40 years, and staff had a short clinical working time (51.0% with work tenure ≤ 10 years). In addition, as their service objects are mainly community and rural residents, general practitioners with comprehensive medical knowledge and skills are the most common (61.4%). The number of people with senior professional titles accounted for only 6.7%, and the number of people with a monthly income of less than 5000 yuan accounted for 82.9%. Compared with staff in Western China, medical staff in the economically developed eastern region were better paid (40.1% with monthly income > 8000). Consistent with the results already reported [15], economically developed regions attract higher quality medical workers by offering better working conditions and higher income, which is one of the influencing factors of the lack of talent in primary hospitals. The current cost of health care in China is rising year by year, but the equity of medical services is poor due to geographical and demographic reasons [16]. Despite the success of the hierarchical diagnosis and treatment system, many problems exist, leading to the unbalanced distribution of medical resources [17]. In addition, we found that there is a significant gender gap among Chinese physicians, with only 30.0% of physicians being women, compared to men, who generally have higher salaries and more opportunities for promotion [18]. The percentage of female medical workers in primary hospitals was 65.1%, but only 5.3% held senior professional titles. Despite the increasing number of women entering the medical field internationally, all countries still show the limitation of underrepresentation of women in higher-level professional titles [19].

In the early treatment of all kinds of critically ill patients, the diagnosis of aetiology is not necessary, and it is more important to provide emergency life support [20, 21]. Studies have shown that patients treated by emergency teams trained in advanced life support have better outcomes [22, 23]. The number and degree of medical staff’s first aid knowledge and skills are closely related to the success rate and cure rate of critically ill patients. We found that medical staff who were in Western China, were aged 31–40, had working tenure > 10 years, were male, were doctors, had a master’s degree or above, worked in the emergency department, and had a senior professional title had better first aid knowledge and skills. Compared with the evaluation of first aid resuscitation performed by hospital staff in Mozambique (average score of 42%) presented by Merchant et al. [24], the first aid ability of Chinese medical staff was more satisfactory (average score of 72.9%). In addition, the first aid ability of medical staff differed across hospitals. The first aid knowledge and skill score of medical staff in tertiary hospitals (209.7 ± 45.0, total score 280) was much higher than that of staff in primary hospitals (157.7 ± 41.1, total score 280). Studies have found that the death rate of patients experiencing cardiac arrest in tertiary hospitals is significantly lower than that of patients admitted to nontertiary hospitals [25]. This may be because the training function for resuscitation care after cardiac arrest is usually performed by tertiary hospitals [26]. Medical staff in high-level hospitals receive regular training to improve their ability to judge and manage various medical conditions and to adopt more aggressive treatment strategies. In contrast, lower-level hospitals are limited in terms of personnel, technology, and equipment and lack training opportunities. As a result, they have a lower first aid capacity and a lower success rate of in-hospital rescue.

The analysis of first aid training needs revealed that primary hospitals had the highest training needs score (240.6 ± 44.0, total score of 280). When asked about the level of need for various first aid knowledge and skills, the majority of primary medical personnel chose “very much needed”. The first aid knowledge of most interest to the primary medical personnel was the emergency management of shock, myocardial infarction, and hypoglycaemia. The first aid skills of interest tended to be the learning of cardiopulmonary resuscitation, airway obstruction removal, and dressing and suturing skills. This differs from the training needs of staff in secondary and tertiary hospitals. Responding to sudden acute and critical events does not require all hospitals to have the same response capabilities; rather, based on ensuring that hospitals at all levels have basic response capabilities, the disease spectrum of local acute patients is used as a reference, and the unique functions and missions of hospitals at different levels are combined to design emergency training appropriate for each to improve the response capabilities of the overall health care system [27].

The results of multiple linear regression analysis showed that the department of medical staff (β = -0.247) was an important predictor of the first aid ability score. Our results are confirmed by Silvia et al. [28], who found that emergency department staff tend to have better emergency competency. Women (β = -0.043) and nonphysicians (β = -0.210) were associated with reduced first aid ability scores, consistent with Nogaro et al. [29]. The geographical location of the hospital (β = 0.157) and the working tenure of the medical staff (β = 0.085) ranked 1st and 2nd, respectively, for their influence on training need scores. The willingness to train is strong in the less-developed areas of Western China [30]. With the growth of working time and the update of first aid knowledge and skills, many medical staff feel that their first aid ability is insufficient and outdated and are eager for guidance regarding standardized operation processes and strengthened proficiency in first aid training. In addition, we found that there was a direct and significant correlation between the total score of first aid ability and the total score of training needs, but there was no correlation between first aid ability and training needs in primary hospitals. This may be due to the generally weak grasp of first aid knowledge and skills of medical workers in primary hospitals, which contradicts the strong training need. It also suggests that the government and relevant departments of health administration should increase the policy targeting and resource input to the construction of primary medical institutions, pay attention to the training of medical personnel’s first aid ability, and improve the current situation of primary medical services.

The World Health Organization recommends that countries develop comprehensive first aid training programs to address acute and critical challenges. A North American study suggests that online learning and simulated training may be good educational methods for practising physicians to maintain knowledge and learn new techniques [31]. In terms of the selection of training methods, we suggest the use of modern technologies, such as situational simulation training, integrated simulation-based medical teaching, and other ways to assist first aid training, ensure patient safety, shorten the training period and enhance the training effect [32, 33]. Considering the current situation in which primary medical workers are limited, a training model should be designed to focus on basic faculty training and carry out training at each level [34, 35]. In addition, it is necessary to give full play to the radiation role of tertiary hospitals’ superior resources, establish online education and learning platforms, and strengthen cooperation and exchanges with secondary and primary medical institutions.

### Advantages and limitations

This study has several advantages. First, we investigated a sample of medical personnel from 12 provinces in China, and the rich sampling scheme and wide range of survey sites make the findings more representative and universal. The diversity of sample features allows for in-depth exploration of the data. In addition, previous studies have mainly assessed the need for emergency training in a single region or a single level of hospital, and our study fills a gap in this area.

The study has some limitations. First, this is a cross-sectional study that cannot draw a causal link, and further longitudinal studies are needed. Second, first aid knowledge involves a wide range of disciplines, and this study can only reflect part of the participants’ first aid level. Data were collected through self-designed questionnaires, and recall bias was inevitable.

## Conclusion

There is a correlation between the first aid knowledge and skills of Chinese medical staff and their training needs. The first aid level is closely related to the background of medical staff. The score of first aid knowledge and skills of medical staff in tertiary hospitals is higher than that of their counterparts in nontertiary hospitals. There is a strong demand for medical staff training in primary hospitals, but the shortage of human resources is serious. Therefore, it is necessary for society and the government to increase the policy and resource support for primary medical institutions in economically underdeveloped areas. It is suggested that hospitals at all levels reasonably set first aid training objectives and contents according to their conditions to improve the overall first aid level of medical staff in China. In addition, intervention studies are needed to further explore the methods and effects of first aid education and training for medical staff.

### Electronic supplementary material

Below is the link to the electronic supplementary material.

Supplementary Material 1

## Data Availability

The datasets used in this study are available from the corresponding author upon request.

## Abbreviations

KMO: Kaiser-Meyer-Olkin
χ^2^: Chi-square test
ANOVA: Analysis of variance
SD: Standard deviation
ARDS: Acute respiratory distress syndrome
CPR: Cardiopulmonary resuscitation
ECG: Electrocardiographic
SE: Standard error
