# Supplementary material for: Current situation and needs analysis of medical staff first aid ability in China: a cross-sectional study
Source: BMC Emerg Med. 2023 Nov 3;23:128. doi: 10.1186/s12873-023-00891-x (PMC10623825; doi:10.1186/s12873-023-00891-x)
Supplement: Supplementary file 1 — Supplementary Material 1 [file 12873_2023_891_MOESM1_ESM.docx]

**Supplementary File 1. Questionnaire**

**Questionnaire of medical staff first aid knowledge and skills and training willingness**

Medical personnel's first-aid ability is critical to improving the quality and success rate of treatment for all types of emergency patients because they are the first line of health defense in dealing with public health emergencies. Teaching medical personnel first aid knowledge and techniques, as well as equipping them with the ability to use them, is a critical factor in saving patients' lives.

**Background**

1. Age : ___ years. ( Please fill in the whole number)

2. The number of years you have been engaged in medical and health work :________ years. ( Please fill in the whole number, more than half a year please fill in 1 year)

3. The location of the medical unit where you currently work :________ province _________ city _________ county/district.

4. Your gender is?

○ Male

○ Female

5. Your occupation type is?

○ Doctor

○ Nurse

○ Other (please specify) _________

6. Your highest degree is?

○ Technical Secondary School Degree

○ Junior College

○ Undergraduate Degree

○ Master's Degree

○ Doctor Degree

1. The level of your hospital?

○ Class A tertiary hospital

○ Class B tertiary hospital

○ Class A secondary hospitals

○ Class B secondary hospitals

○ Primary hospital

8. Your department?

○ Emergency Department

○ General Surgery

○ General Medicine

○ Other (please specify) _________

9. Your title level is?

○ No job title

○ Primary title

○ Middle title

○ Vice-senior title

○ Senior title

10. Your personal average monthly income is approximately?

○ < 3000 yuan

○ 3000 - 5000 yuan

○ 5001 - 8000 yuan

○ 8001 - 12000 yuan

○ > 12000 yuan

**Scale**

1. First aid theoretical knowledge and training needs. (please mark √ in □ and fill in the information as required)

| ****Variables**** | | **Knowledge mastery** | | | | | **Theoretical training intention** | | | | |
| --- | --- | --- | --- | --- | --- | --- | --- | --- | --- | --- | --- |
|  |  | **Proficient mastery** | **Able to understand**  **General** | **General** | **Relatively unfamiliar** | **Unfamiliar** | **Be in great need** | **Comparative need** | **In general** | **Not much need** | **Don't need** |
| First aid overview | |  |  |  |  |  |  |  |  |  |  |
| Inspection classification | |  |  |  |  |  |  |  |  |  |  |
| Emergency trauma | Emergency management of head trauma |  |  |  |  |  |  |  |  |  |  |
|  | Emergency treatment for chest trauma |  |  |  |  |  |  |  |  |  |  |
|  | Emergency management of abdominal trauma |  |  |  |  |  |  |  |  |  |  |
|  | Emergency treatment for trauma to extremities |  |  |  |  |  |  |  |  |  |  |
|  | Emergency treatment of spinal trauma |  |  |  |  |  |  |  |  |  |  |
| Environmental emergency | Early treatment of burns |  |  |  |  |  |  |  |  |  |  |
|  | Early treatment of frostbite |  |  |  |  |  |  |  |  |  |  |
|  | Early treatment of drowning |  |  |  |  |  |  |  |  |  |  |
| Circulating emergency | Emergency management of shock |  |  |  |  |  |  |  |  |  |  |
|  | Emergency management of arrhythmia |  |  |  |  |  |  |  |  |  |  |
|  | Emergency management of acute left heart failure |  |  |  |  |  |  |  |  |  |  |
|  | Emergency management of myocardial infarction |  |  |  |  |  |  |  |  |  |  |
|  | Emergency management of hypertensive crisis |  |  |  |  |  |  |  |  |  |  |
| Respiratory emergency | Emergency management of ARDS |  |  |  |  |  |  |  |  |  |  |
|  | Emergency management of pulmonary embolism |  |  |  |  |  |  |  |  |  |  |
|  | Emergency management of severe asthma |  |  |  |  |  |  |  |  |  |  |
|  | Emergency management of respiratory failure |  |  |  |  |  |  |  |  |  |  |
| Gastrointestinal emergency | Emergency management of intestinal obstruction |  |  |  |  |  |  |  |  |  |  |
|  | Emergency management of acute cholangitis |  |  |  |  |  |  |  |  |  |  |
|  | Emergency management of gastrointestinal bleeding |  |  |  |  |  |  |  |  |  |  |
|  | Emergency management of severe pancreatitis |  |  |  |  |  |  |  |  |  |  |
| Endocrine emergency | Emergency management of hypoglycemia |  |  |  |  |  |  |  |  |  |  |
|  | Emergency management of thyroid crisis |  |  |  |  |  |  |  |  |  |  |
|  | Emergency management of hypertonic coma |  |  |  |  |  |  |  |  |  |  |
|  | Emergency management of diabetic ketoacidosis |  |  |  |  |  |  |  |  |  |  |
| Nervous system emergency | Emergency management of epilepsy |  |  |  |  |  |  |  |  |  |  |
|  | Emergency management of cerebral embolism |  |  |  |  |  |  |  |  |  |  |
|  | Emergency management of cerebral hemorrhage |  |  |  |  |  |  |  |  |  |  |
|  | Emergency management of high cranial pressure |  |  |  |  |  |  |  |  |  |  |
|  | Emergency management of transient ischemic attack |  |  |  |  |  |  |  |  |  |  |
| Other emergency | Emergency management of ectopic pregnancy |  |  |  |  |  |  |  |  |  |  |
|  | Emergency management of anaphylaxis |  |  |  |  |  |  |  |  |  |  |
|  | Emergency management of transfusion reactions |  |  |  |  |  |  |  |  |  |  |
|  | Emergency management of multiple organ failure |  |  |  |  |  |  |  |  |  |  |
|  | Emergency management of water, electrolyte and acid-base imbalance |  |  |  |  |  |  |  |  |  |  |

1. Individual first aid skills and training needs. (please mark √ in □ and fill in the information as required)

| ****Variables**** | **Knowledge mastery** | | | | | **Theoretical training intention** | | | | |
| --- | --- | --- | --- | --- | --- | --- | --- | --- | --- | --- |
|  | **Proficient mastery** | **Able to understand**  **General** | **General** | **Relatively unfamiliar** | **Unfamiliar** | **Be in great need** | **Comparative need** | **In general** | **Not much need** | **Don't need** |
| Adult CPR |  |  |  |  |  |  |  |  |  |  |
| Infant CPR |  |  |  |  |  |  |  |  |  |  |
| Exclusion of adult airway foreign body obstruction |  |  |  |  |  |  |  |  |  |  |
| Exclusion of infant airway foreign body obstruction |  |  |  |  |  |  |  |  |  |  |
| Adult tracheal intubation |  |  |  |  |  |  |  |  |  |  |
| Cricothyroid puncture |  |  |  |  |  |  |  |  |  |  |
| Electrical defibrillation |  |  |  |  |  |  |  |  |  |  |
| Electric compounding |  |  |  |  |  |  |  |  |  |  |
| Deep vein puncture |  |  |  |  |  |  |  |  |  |  |
| Thoracentesis |  |  |  |  |  |  |  |  |  |  |
| Abdominal puncture |  |  |  |  |  |  |  |  |  |  |
| Lumbar puncture |  |  |  |  |  |  |  |  |  |  |
| Bone marrow aspiration |  |  |  |  |  |  |  |  |  |  |
| Closed thoracic drainage |  |  |  |  |  |  |  |  |  |  |
| Pericardiocentesis |  |  |  |  |  |  |  |  |  |  |
| War creation technology |  |  |  |  |  |  |  |  |  |  |
| Dressing change, clear planing and suture |  |  |  |  |  |  |  |  |  |  |
| Arterial puncture |  |  |  |  |  |  |  |  |  |  |
| ECG monitoring and recognition |  |  |  |  |  |  |  |  |  |  |

**Supplementary File 2. Table**

**Supplementary Table 1** Correlation analysis of general information and first aid knowledge and skill scores of Chinese medical personnel (China, 2022-2023).

| **Variables** | **Scores (mean±SD)** | **Statistical values (F)** | **P value** | **Kendall’s tau-b** | **P value** |
| --- | --- | --- | --- | --- | --- |
| Geographic region |  |  |  |  |  |
| Eastern China | 196.42±50.22 | 150.18 | < 0.001 | 0.026 | < 0.001 |
| Central China | 183.36±48.52 |  |  |  |  |
| Western China | 199.06±50.91 |  |  |  |  |
| Age |  |  |  |  |  |
| ≤30 | 183.34±46.74 | 152.55 | < 0.001 | 0.094 | < 0.001 |
| 31-40 | 198.92±50.18 |  |  |  |  |
| ≥41 | 195.59±53.56 |  |  |  |  |
| Work tenure |  |  |  |  |  |
| ≤10 | 186.82±48.79 | 227.36 | < 0.001 | 0.107 | < 0.001 |
| >10 | 199.52±51.37 |  |  |  |  |
| Sex |  |  |  |  |  |
| Male | 206.69±49.50 | 928.83 | < 0.001 | -0.205 | < 0.001 |
| Female | 181.65±48.22 |  |  |  |  |
| Job title |  |  |  |  |  |
| Doctors | 204.25±48.85 | 1090.08 | < 0.001 | -0.235 | < 0.001 |
| Nures | 186.19±43.27 |  |  |  |  |
| Others | 121.71±49.19 |  |  |  |  |
| Education level |  |  |  |  |  |
| Associate’s degree or vocational diploma | 172.27±48.66 | 557.88 | < 0.001 | 0.205 | < 0.001 |
| Bachelor degree | 200.63±48.98 |  |  |  |  |
| Master degree or higher | 205.87±43.34 |  |  |  |  |
| Department |  |  |  |  |  |
| Emergency | 215.63±42.60 | 1049.17 | < 0.001 | -0.314 | < 0.001 |
| general surgery | 187.19±39.56 |  |  |  |  |
| General medicine | 172.65±43.21 |  |  |  |  |
| Others | 170.95±51.57 |  |  |  |  |
| Professional title |  |  |  |  |  |
| Elementary or below | 181.82±49.50 | 467.375 | < 0.001 | 0.194 | < 0.001 |
| Intermediate | 200.44±48.60 |  |  |  |  |
| Senior | 214.60±46.35 |  |  |  |  |
| Annual personal income (¥) |  |  |  |  |  |
| <3000 | 160.08±48.15 | 752.35 | < 0.001 | 0.280 | < 0.001 |
| 3000-5000 | 181.38±48.33 |  |  |  |  |
| 5001-8000 | 201.30±46.71 |  |  |  |  |
| ≥8001 | 218.28±43.67 |  |  |  |  |
| Hospital level |  |  |  |  |  |
| Tertiary | 209.74±45.04 | 1170.31 | < 0.001 | -0.280 | < 0.001 |
| Secondary | 189.96±48.28 |  |  |  |  |
| Primary | 157.72±47.14 |  |  |  |  |

**Supplementary Table 2** First aid training needs (China, 2022-2023).

| **Variables** | **Don't need** | **Not much need** | **In general** | **Comparative need** | **Be in great need** |
| --- | --- | --- | --- | --- | --- |
| First aid knowledge |  |  |  |  |  |
| First aid overview | 514(3.78) | 552(4.05) | 2117(15.55) | 5248(38.55) | 5182(38.07) |
| Inspection classification | 515(3.78) | 622(4.57) | 2046(15.03) | 5326(39.12) | 5104(37.49) |
| Emergency management of head trauma | 501(3.68) | 494(3.63) | 2062(15.15) | 5250(38.57) | 5306(38.98) |
| Emergency treatment for chest trauma | 506(3.72) | 561(4.12) | 1994(14.65) | 5229(38.41) | 5323(39.1) |
| Emergency management of abdominal trauma | 471(3.46) | 463(3.4) | 2012(14.78) | 5297(38.91) | 5370(39.45) |
| Emergency treatment for trauma to extremities | 456(3.35) | 535(3.93) | 2004(14.72) | 5244(38.52) | 5374(39.48) |
| Emergency treatment of spinal trauma | 463(3.4) | 486(3.57) | 2089(15.35) | 5273(38.74) | 5302(38.95) |
| Early treatment of burns | 467(3.43) | 481(3.53) | 1984(14.57) | 5281(38.79) | 5400(39.67) |
| Early treatment of frostbite | 455(3.34) | 470(3.45) | 1967(14.45) | 5337(39.21) | 5384(39.55) |
| Early treatment of drowning | 450(3.31) | 497(3.65) | 1884(13.84) | 5184(38.08) | 5598(41.12) |
| Emergency management of shock | 457(3.36) | 427(3.14) | 1849(13.58) | 5137(37.74) | 5743(42.19) |
| Emergency management of arrhythmia | 441(3.24) | 454(3.34) | 1879(13.8) | 5134(37.71) | 5705(41.91) |
| Emergency management of acute left heart failure | 456(3.35) | 439(3.22) | 1896(13.93) | 5139(37.75) | 5683(41.75) |
| Emergency management of myocardial infarction | 442(3.25) | 473(3.47) | 1903(13.98) | 5073(37.27) | 5722(42.03) |
| Emergency management of hypertensive crisis | 443(3.25) | 405(2.98) | 1959(14.39) | 5127(37.66) | 5679(41.72) |
| Emergency management of ARDS | 448(3.29) | 447(3.28) | 1940(14.25) | 5103(37.49) | 5675(41.69) |
| Emergency management of pulmonary embolism | 433(3.18) | 432(3.17) | 1891(13.89) | 5167(37.96) | 5690(41.8) |
| Emergency management of severe asthma | 414(3.04) | 482(3.54) | 1934(14.21) | 5091(37.4) | 5692(41.81) |
| Emergency management of respiratory failure | 418(3.07) | 416(3.06) | 1955(14.36) | 5129(37.68) | 5695(41.84) |
| Emergency management of intestinal obstruction | 440(3.23) | 427(3.14) | 1997(14.67) | 5105(37.5) | 5644(41.46) |
| Emergency management of acute cholangitis | 442(3.25) | 426(3.13) | 1959(14.39) | 5245(38.53) | 5541(40.7) |
| Emergency management of gastrointestinal bleeding | 432(3.17) | 469(3.45) | 1907(14.01) | 5112(37.55) | 5693(41.82) |
| Emergency management of severe pancreatitis | 431(3.17) | 414(3.04) | 1998(14.68) | 5132(37.7) | 5638(41.42) |
| Emergency management of hypoglycemia | 446(3.28) | 425(3.12) | 1954(14.35) | 5045(37.06) | 5743(42.19) |
| Emergency management of thyroid crisis | 420(3.09) | 417(3.06) | 1958(14.38) | 5203(38.22) | 5615(41.25) |
| Emergency management of hypertonic coma | 432(3.17) | 452(3.32) | 2002(14.71) | 5131(37.69) | 5596(41.11) |
| Emergency management of diabetic ketoacidosis | 414(3.04) | 419(3.08) | 2021(14.85) | 5130(37.68) | 5629(41.35) |
| Emergency management of epilepsy | 427(3.14) | 432(3.17) | 1966(14.44) | 5158(37.89) | 5630(41.36) |
| Emergency management of cerebral embolism | 427(3.14) | 404(2.97) | 1893(13.91) | 5227(38.4) | 5662(41.59) |
| Emergency management of cerebral hemorrhage | 409(3) | 449(3.3) | 1902(13.97) | 5180(38.05) | 5673(41.67) |
| Emergency management of high cranial pressure | 414(3.04) | 398(2.92) | 1995(14.66) | 5150(37.83) | 5656(41.55) |
| Emergency management of transient ischemic attack | 395(2.9) | 426(3.13) | 1973(14.49) | 5166(37.95) | 5653(41.53) |
| Emergency management of ectopic pregnancy | 430(3.16) | 469(3.45) | 2186(16.06) | 5088(37.38) | 5440(39.96) |
| Emergency management of anaphylaxis | 408(3) | 446(3.28) | 1903(13.98) | 5060(37.17) | 5796(42.58) |
| Emergency management of transfusion reactions | 422(3.1) | 384(2.82) | 1986(14.59) | 5108(37.52) | 5713(41.97) |
| Emergency management of multiple organ failure | 421(3.09) | 411(3.02) | 1938(14.24) | 5127(37.66) | 5716(41.99) |
| Emergency management of water, electrolyte and acid-base imbalance | 419(3.08) | 382(2.81) | 1986(14.59) | 5095(37.43) | 5731(42.1) |
| Single first aid skill |  |  |  |  |  |
| Adult CPR | 519(3.81) | 491(3.61) | 2015(14.8) | 4318(31.72) | 6270(46.06) |
| Infant CPR | 465(3.42) | 554(4.07) | 2009(14.76) | 4461(32.77) | 6124(44.99) |
| Exclusion of adult airway foreign body obstruction | 478(3.51) | 450(3.31) | 2005(14.73) | 4448(32.67) | 6232(45.78) |
| Exclusion of infant airway foreign body obstruction | 469(3.45) | 485(3.56) | 2061(15.14) | 4478(32.9) | 6120(44.96) |
| Adult tracheal intubation | 457(3.36) | 526(3.86) | 2153(15.82) | 4596(33.76) | 5881(43.2) |
| Cricothyroid puncture | 416(3.06) | 590(4.33) | 2268(16.66) | 4581(33.65) | 5758(42.3) |
| Electrical defibrillation | 435(3.2) | 467(3.43) | 2144(15.75) | 4554(33.45) | 6013(44.17) |
| Electric compounding | 435(3.2) | 477(3.5) | 2142(15.73) | 4616(33.91) | 5943(43.66) |
| Deep vein puncture | 416(3.06) | 502(3.69) | 2215(16.27) | 4681(34.39) | 5799(42.6) |
| Pleural puncture | 437(3.21) | 618(4.54) | 2361(17.34) | 4580(33.64) | 5617(41.26) |
| Abdominal puncture | 457(3.36) | 540(3.97) | 2420(17.78) | 4566(33.54) | 5630(41.36) |
| Lumbar puncture | 451(3.31) | 575(4.22) | 2418(17.76) | 4570(33.57) | 5599(41.13) |
| Bone marrow aspiration | 468(3.44) | 585(4.3) | 2404(17.66) | 4672(34.32) | 5484(40.29) |
| Closed thoracic drainage | 456(3.35) | 598(4.39) | 2373(17.43) | 4614(33.89) | 5572(40.93) |
| pericardiocentesis | 452(3.32) | 578(4.25) | 2404(17.66) | 4607(33.84) | 5572(40.93) |
| Trauma techniques | 425(3.12) | 455(3.34) | 2115(15.54) | 4541(33.36) | 6077(44.64) |
| Dressing change, debridement and suture | 418(3.07) | 486(3.57) | 2194(16.12) | 4538(33.34) | 5977(43.91) |
| Arterial puncture | 455(3.34) | 528(3.88) | 2277(16.73) | 4565(33.53) | 5788(42.52) |
| Ecg monitoring ECG recognition | 399(2.93) | 416(3.06) | 2124(15.6) | 4555(33.46) | 6119(44.95) |

**Supplementary Table 3** First aid training needs of a primary hospital (China, 2022-2023).

| Variables | Primary hospital n（%） | | | | |
| --- | --- | --- | --- | --- | --- |
|  | Don't need | Not much need | In general | Comparative need | Be in great need |
| First aid knowledge |  |  |  |  |  |
| First aid overview | 67（2.5） | 63（2.4） | 216（8.2） | 1021（38.6） | 1281（48.4） |
| Inspection classification | 63（2.4） | 67（2.5） | 232（8.8） | 1054（39.8） | 1232（46.5） |
| Emergency management of head trauma | 65（2.5） | 62（2.3） | 224（8.5） | 1029（38.9） | 1268（47.9） |
| Emergency treatment for chest trauma | 68（2.6） | 62（2.3） | 222（8.4） | 1032（39.0） | 1264（47.7） |
| Emergency management of abdominal trauma | 65（2.5） | 68（2.6） | 207（7.8） | 1043（39.4） | 1265（47.8） |
| Emergency treatment for trauma to extremities | 64（2.4） | 61（2.3） | 212（8.0） | 1019（38.5） | 1292（48.8） |
| Emergency treatment of spinal trauma | 69（2.6） | 66（2.5） | 212（8.0） | 1037（39.2） | 1264（47.7） |
| Early treatment of burns | 65（2.5） | 63（2.4） | 201（7.6） | 1026（38.7） | 1293（48.8） |
| Early treatment of frostbite | 62（2.3） | 61（2.3） | 203（7.7） | 1029（38.9） | 1293（48.8） |
| Early treatment of drowning | 52（2.0） | 64（2.4） | 192（7.3） | 995（37.6） | 1345（50.8） |
| Emergency management of shock | 64（2.4） | 57（2.2） | 185（7.0） | 967（36.5） | 1375（51.9） |
| Emergency management of arrhythmia | 58（2.2） | 59（2.2） | 198（7.5） | 984（37.2） | 1349（50.9） |
| Emergency management of acute left heart failure | 60（2.3） | 60（2.3） | 201（7.6） | 1002（37.8） | 1325（50.0） |
| Emergency management of myocardial infarction | 56（2.1） | 58（2.2） | 198（7.5） | 986（37.2） | 1350（51.0） |
| Emergency management of hypertensive crisis | 58（2.2） | 65（2.5） | 189（7.1） | 993（37.5） | 1343（50.7） |
| Emergency management of ARDS | 61（2.3） | 59（2.2） | 203（7.7） | 1009（38.1） | 1316（49.7） |
| Emergency management of pulmonary embolism | 55（2.1） | 65（2.5） | 215（8.1） | 1015（38.3） | 1298（49.0） |
| Emergency management of severe asthma | 52（2.0） | 69（2.6） | 198（7.5） | 989（37.3） | 1340（50.6） |
| Emergency management of respiratory failure | 59（2.2） | 56（2.1） | 203（7.7） | 1012（38.2） | 1318（49.8） |
| Emergency management of intestinal obstruction | 57（2.2） | 58（2.2） | 205（7.7） | 1001（37.8） | 1327（50.1） |
| Emergency management of acute cholangitis | 57（2.2） | 61（2.3） | 203（7.7） | 1017（38.4） | 13110（49.5） |
| Emergency management of gastrointestinal bleeding | 60（2.3） | 61（2.3） | 194（7.3） | 999（37.4） | 1342（50.7） |
| Emergency management of severe pancreatitis | 55（2.1） | 63（2.4） | 221（8.3） | 998（37.7） | 1311（49.5） |
| Emergency management of hypoglycemia | 60（2.3） | 58（2.2） | 200（7.6） | 970（36.6） | 1360（51.4） |
| Emergency management of thyroid crisis | 62（2.3） | 62（2.3） | 229（8.6） | 999（37.7） | 1296（48.9） |
| Emergency management of hypertonic coma | 63（2.4） | 60（2.3） | 219（8.3） | 1000（37.8） | 1306（49.3） |
| Emergency management of diabetic ketoacidosis | 60（2.3） | 68（2.6） | 208（7.9） | 996（37.6） | 1316（49.7） |
| Emergency management of epilepsy | 63（2.4） | 61（2.3） | 201（7.6） | 999（37.7） | 1324（50.0） |
| Emergency management of cerebral embolism | 61（2.3） | 58（2.2） | 210（7.9） | 1002（37.8） | 1317（49.7） |
| Emergency management of cerebral hemorrhage | 65（2.5） | 64（2.4） | 196（7.4） | 1005（38.0） | 1318（49.8） |
| Emergency management of high cranial pressure | 61（2.3） | 66（2.5） | 195（7.4） | 1012（38.2） | 1314（49.6） |
| Emergency management of transient ischemic attack | 54（2.0） | 67（2.5） | 189（7.1） | 1011（38.2） | 1327（50.1） |
| Emergency management of ectopic pregnancy | 65（2.5） | 61（2.3） | 230（8.7） | 985（37.2） | 1307（49.4） |
| Emergency management of anaphylaxis | 59（2.2） | 54（2.0） | 179（6.8） | 963（36.4） | 1393（52.6） |
| Emergency management of transfusion reactions | 66（2.5） | 62（2.3） | 210（7.9） | 977（36.9） | 1333（50.3） |
| Emergency management of multiple organ failure | 63（2.4） | 60（2.3） | 216（8.2） | 967（36.5） | 1342（50.7） |
| Emergency management of water, electrolyte and acid-base imbalance | 65（2.5） | 50（1.9） | 208（7.9） | 970（36.6） | 1355（51.2） |
| Single first aid skill |  |  |  |  |  |
| Adult CPR | 56（2.1） | 46（1.7） | 212（8.0） | 860（32.5） | 1474（55.7） |
| Infant CPR | 64（2.4） | 51（1.9） | 205（7.7） | 896（33.8） | 1432（54.1） |
| Exclusion of adult airway foreign body obstruction | 67（2.5） | 49（1.9） | 194（7.3） | 886（33.5） | 1452（54.8） |
| Exclusion of infant airway foreign body obstruction | 66（2.5） | 51（1.9） | 206（7.8） | 887（33.5） | 1438（54.3） |
| Adult tracheal intubation | 65（2.5） | 90（3.4） | 253（9.6） | 896（33.8） | 1344（50.8） |
| Cricothyroid puncture | 62（2.3） | 91（3.4） | 303（11.4） | 878（33.2） | 1314（49.6） |
| Electrical defibrillation | 56（2.1） | 74（2.8） | 258（99.7） | 903（34.1） | 1357（51.2） |
| Electric compounding | 58（2.2） | 70（2.6） | 278（10.5） | 903（34.1） | 1339（50.6） |
| Deep vein puncture | 59（2.2） | 86（3.2） | 284（10.7） | 909（34.3） | 1310（49.5） |
| Pleural puncture | 63（2.4） | 95（3.6） | 298（11.3） | 900（34.0） | 1292（48.8） |
| Abdominal puncture | 63（2.4） | 92（3.5） | 296（11.2） | 896（33.8） | 1301（49.1） |
| Lumbar puncture | 65（2.5） | 98（3.7） | 305（11.5） | 894（33.8） | 1286（48.6） |
| Bone marrow aspiration | 73（2.8） | 103（3.9） | 324（12.2） | 886（33.5） | 1262（47.7） |
| Closed thoracic drainage | 63（2.4） | 97（3.7） | 301（11.4） | 900（34.0） | 1287（48.6） |
| pericardiocentesis | 66（2.5） | 110（4.2） | 318（12.0） | 894（33.8） | 1260（47.6） |
| Trauma techniques | 54（2.0） | 63（2.4） | 232（8.8） | 887（33.5） | 1412（53.3） |
| Dressing change, debridement and suture | 43（1.6） | 62（2.3） | 226（8.5） | 890（33.6） | 1427（53.9） |
| Arterial puncture | 66（2.5） | 81（3.1） | 286（10.8） | 899（34.0） | 1316（49.7） |
| Ecg monitoring ECG recognition | 52（2.0） | 57（2.2） | 223（8.4） | 912（34.4） | 1404（53.0） |
